# Oral Cancer Stem Cell-Derived Small Extracellular Vesicles Promote M2 Macrophage Polarization and Suppress CD4^+^ T-Cell Activity by Transferring UCA1 and Targeting LAMC2

**DOI:** 10.1155/2022/5817684

**Published:** 2022-11-28

**Authors:** Lan Wu, Sai Ye, Yilin Yao, Chenping Zhang, Wei Liu

**Affiliations:** ^1^Department of Oral Mucosal Diseases, Shanghai Ninth People's Hospital, Shanghai Jiao Tong University School of Medicine, Shanghai, China; ^2^College of Stomatology, Shanghai Jiao Tong University, National Center for Stomatology, China; ^3^National Clinical Research Center for Oral Diseases, Shanghai Key Laboratory of Stomatology, Shanghai, China; ^4^Department of Oral Maxillofacial-Head and Neck Oncology, Shanghai Ninth People's Hospital, Shanghai Jiao Tong University School of Medicine, Shanghai, China

## Abstract

Cancer-derived small extracellular vesicles (sEVs) are emerging as crucial mediators of intercellular communication between cancer cells and M2-tumor-associated macrophages (M2-TAMs) via transferring lncRNAs. We previously reported that miR-134 blocks the expression of its targeting protein LAMC2 via the PI3K/AKT pathway and inhibits cancer stem cell (CSC) migration and invasion in oral squamous cell carcinoma (OSCC). This study hypothesize that OSCC-CSC-derived small extracellular vesicles (OSCC-CSC-sEVs) transfer a ceRNA of miR-134 and consequently promote M2 macrophage polarization by targeting LAMC2 via the PI3K/AKT pathway through *in vitro* and *in vivo* experiment methods. The results showed that sEVs derived from CD133^+^CD44^+^ OSCC cells promoted M2 polarization of macrophages by detecting several M2 macrophage markers (CD163, IL-10, Arg-1, and CD206^+^CD11b^+^). Mechanistically, we revealed that the lncRNA UCA1, by binding to miR-134, modulated the PI3K/AKT pathway in macrophages via targeting LAMC2. Importantly, OSCC-CSC-sEV transfer of UCA1, by targeting LAMC2, promoted M2 macrophage polarization and inhibited CD4^+^ T-cell proliferation and IFN-*γ* production *in vitro* and *in vivo*. Functionally, we demonstrated that M2-TAMs, by transferring exosomal UCA and consequently targeting LAMC2, enhanced cell migration and invasion of OSCC *in vitro* and the tumorigenicity of OSCC xenograft in nude mice. In conclusion, our results indicated that OSCC-CSC-sEV transfer of UCA1 promotes M2 macrophage polarization via a LAMC2-mediated PI3K/AKT axis, thus facilitating tumor progression and immunosuppression. Our findings provide a new understanding of OSCC-CSC molecular mechanisms and suggest a potential therapeutic strategy for OSCC through targeting CSC-sEVs and M2-TAMs.

## 1. Introduction

The tumor microenvironment provides survival conditions that may enable tumor growth and progression [1]. It is composed of tumor cells and a variety of stromal cells, including matrix immune cells, among which mononuclear cells or macrophages are an abundant and important component [2, 3]. Under different regulatory mechanisms, macrophages can differentiate into two main subtypes: the classically activated M1 subtype exhibiting antitumor immunity and inflammatory responses and the alternatively activated M2 subtype exhibiting a protumor effect that contributes to tumor development and progression [4, 5]. Tumor-associated macrophages (TAMs) are a macrophage population recruited and educated by cancer cells [2, 3]. TAMs are typically maintained in an M2-polarized condition with a protumor phenotype involving remodeling of the extracellular matrix, immunosuppression, and tumor progression in the tumor microenvironment [5].

Small extracellular vesicles (sEVs) including exosomes act as nanoscale messengers. sEVs have emerged as crucial mediators of intercellular communication between cancer cells and stromal cells in the tumor microenvironment and have been found to function by transferring cargos, including proteins and RNAs [6, 7]. Recently, studies have shown that cancer-derived sEVs promote M2 macrophage polarization through immune signaling pathways by transferring noncoding RNAs in various cancers. For instance, oral cancer-derived exosomes promote M2 macrophage polarization, mediated by exosome-enclosed miR-29a [8]. Cancer stem cells (CSCs), a small subpopulation of cancer cells, can be identified and isolated according to their expression of distinctive markers. CSC-derived- sEVs are reported to be responsible for disease progression of various cancers [9–11]. Interestingly, CSCs secrete sEVs associated with an immunosuppressive microenvironment and further promote M2 macrophage polarization in glioblastoma [12] and colon cancer [13]. However, whether CSC-derived sEVs transfer lncRNAs that promote M2 macrophage polarization has rarely been reported.

Oral squamous cell carcinoma (OSCC) accounts for more than 90% of all oral cancers and remains a major cause of cancer morbidity and mortality worldwide [14]. We previously reported that miR-134 blocks the expression of its targeting protein LAMC2 via the PI3K/AKT signaling pathway and inhibits CSC migration and invasion in OSCC [15]. We have observed that both sEVs and M2 macrophages exert their biological functions via the PI3K/AKT signaling pathway in various cancers [16–20]. Furthermore, recent reports have shown that LAMC2 induces the infiltration of macrophages in lung cancer [21] and that sEVs transfer a miRNA in ovarian cancer via a LAMC2-mediated PI3K/AKT axis [22]. On the basis of previous studies, we hypothesized that OSCC-CSC-derived sEVs (OSCC-CSC-sEVs) might transfer a ceRNA of miR-134, thereby promoting M2 macrophage polarization by targeting LAMC2 via the PI3K/AKT signaling pathway. As expected, our results revealed that OSCC-CSC-sEVs transferring the lncRNA UCA1 promoted M2 macrophage polarization via a LAMC2-mediated PI3K/AKT axis and further modulated immunosuppression, partly by inhibiting CD4^+^ T-cell proliferation and IFN-*γ* production.

## 2. Materials and Methods

### 2.1. Cell Culture and Identification of OSCC-CSCs

The OSCC cell line Cal27 (CL-0265, Procell, Wuhan, Hubei, China), human oral epithelial cells (HOEC, BS-C00865936, Shanghai Binsui Biotechnology Co., Ltd., Shanghai, China), and HEK-293T cells (CL-0005, Procell) were cultured in Dulbecco's modified Eagle's medium (DMEM) (PM150210A, Procell) containing 10% fetal bovine serum (FBS, 15950-017, Beijing ZEPING Bioscience & Technologies Co., Ltd., Beijing, China), 100 U/mL penicillin, and 100 U/mL streptomycin. Human THP-1 monocytes (CL-0233, Procell) were cultured in Roswell Park Memorial Institute- (RPMI-) 1640 complete medium (PM150110B, Procell) containing 10% FBS, 100 U/mL penicillin, and 100 U/mL streptomycin. All cells were cultured in an incubator at 37°C under 5% CO_2_.

CD133^+^CD44^+^ cells from Cal27 cells were selected as OSCC-CSCs with a magnetic-activated cell sorting system (130-092-545, Miltenyi Biotec GmbH, Bergisch Gladbach, Germany). OSCC-CSCs were identified on the basis of immunofluorescence and sphere formation. Cell sorting and immunofluorescence were performed in accordance with the manufacturer's instructions, as described in our previous study [15].

### 2.2. Sphere Formation Assays

Cal27-CSCs were cultured in serum-free tumorsphere medium DMEM/F-12 (#12500-062, Gibco, Carlsbad, California, USA) supplemented with N-2 additive (#17502048, Gibco), 20 ng/mL recombinant basic fibroblast growth factor (bFGF, #100-18B, PeproTech, Rocky Hill, NJ, USA), and 20 ng/mL epidermal growth factor (EGF, #AF-100-15, PeproTech). Cells were cultured at a density of 7.5 × 10^4^ cells/10 mm dish, and the medium was changed every other day until clonospheres formed.

Cells were transduced according to the instructions for lentiviral infection (Supplementary Table S1) in the following groups: overexpression- (oe-) negative control (NC) lentivirus, oe-UCA1 lentivirus, oe-NC lentivirus+sh-NC lentivirus, oe-UCA1 lentivirus+sh-NC lentivirus, and oe-UCA1 lentivirus+sh-LAMC2 lentivirus. Silencing lentivirus particles was packaged by insertion of the core plasmid (PLKO.1) and auxiliary plasmid (RRE, REV, and Vsvg) of the target gene silencing sequence. The overexpression lentivirus was packaged by insertion of the core plasmid (Fugw-GFP and Plx304) and auxiliary plasmid (RRE, REV, and Vsvg) of the cDNA sequence of the target gene. Lentiviruses were purchased from Genomeditech (Shanghai, China). Primer sequences and plasmid construction were performed by Genomeditech. All experimental steps were implemented according to the manufacturer's instructions.

### 2.3. Isolation and Identification of CSC-Derived sEVs

sEVs were isolated from the supernatants of Cal27-CSCs and Cal27 cells by differential centrifugation. The collected culture supernatant was centrifuged at 300 × *g* for 10 minutes, at 2000 × *g* for 10 minutes, and at 10000 × *g* for 30 minutes. The supernatant was then ultracentrifuged at 110000 × *g* for 2 hours to obtain the precipitate containing sEVs. The collected sEVs were resuspended in PBS. The precipitate was filtered with a 0.22 filter to remove small cell debris, resuspended in PBS, and then ultracentrifuged again for 2 hours at 110000 × *g* to remove the PBS. The sEVs were stored at −80°C before use. The above centrifugation was performed at 4°C. The cells used for sEVs isolation were cultured in medium without sEVs and serum (C38010050, VivaCell, Shanghai, China). The morphology of CSC-derived sEVs was verified with a transmission electron microscope (TEM, H-7650, Hitachi Co., Ltd., Tokyo, Japan) and nanoparticle tracking analysis (NTA, NanoSight LM10 instrument, NanoSight Ltd., Minton Park, UK). Qualitative detection of sEVs was performed on the basis of sEVs surface marker proteins through Western blot analysis.

### 2.4. Macrophage (M*φ*) Induction and Cal27-CSC-sEV Uptake by M*φ*

#### 2.4.1. M*φ* Induction

After centrifugation, the density of logarithmically growing THP-1 cells was adjusted to 2.5 × 10^5^ cells/mL. THP-1 cells were stimulated with 100 ng/mL phorbol 12-myristate 13-acetate (PMA, HY-18739, MedChemExpress, New Jersey, USA). M*φ* macrophages were obtained after incubation in the dark for 48 hours. To detect the effects of Cal27-CSC-derived sEVs (Cal27-CSC-sEVs) on macrophage polarization, we cocultured 20 *μ*g/mL Cal27-CSC-sEVs and M*φ* for 24 hours and then performed measurements.

#### 2.4.2. Cal27-CSC-sEV Uptake by M*φ*

Cal27-CSC-sEVs were exposed to 2 *μ*m PHK67 (MINI67, Sigma-Aldrich, St Louis, MO, USA) fluorescence labeling dye according to the manufacturer's instructions. Briefly, Cal27-CSC-sEVs were labeled with PHK67 on slides, which were soaked in 4% paraformaldehyde for 30 minutes and permeabilized with 2% Triton X-100 for 15 minutes. Afterward, the slides were blocked with 2% BSA for 45 minutes. After staining with 4′,6-diamidino-2-phenylindole (2 *μ*g/mL, C1005, Beyotime), the slides were sealed. Fluorescence expression was detected with a confocal fluorescence microscope.

### 2.5. Bioinformatics Analysis

The Gene Expression Omnibus (https://www.ncbi.nlm.nih.gov/gds) database was used to download the dataset GSE146483, encompassing three normal tissues and eight OSCC tissues. The differentially expressed genes were screened with the R “limma” package (http://www.bioconductor.org/packages/release/bioc/html/limma.html) with |log_2_ fold change (*FC*)| > 2 and *p* < 0.01 as the threshold. The lncRNAs binding the target miRNA were predicted with the RNAInter database (http://www.rna-society.org/rnainter/). The Gene Expression Profiling Interactive Analysis (GEPIA, http://gepia2.cancer-pku.cn/#index) database was used to analyze the differential expression of target genes in The Cancer Genome Atlas (TCGA) dataset. The correlation of gene expression in the TCGA dataset was analyzed with the starBase database (http://Starbase.sysu.edu.cn/index.php).

### 2.6. Dual-Luciferase Reporter Assays

The miR-134 binding site (wild type [WT] or mutant [MUT]) in the UCA1 sequence was inserted into a luciferase reporter vector pmirGLO (3577193, BioVector, Beijing, China) to construct the reporter plasmids pmirGLO-UCA1-WT and pmirGLO-UCA1-MUT. Subsequently, 2.5 *μ*g reporter plasmids were cotransfected with miR-134 mimic plasmid and NC plasmid into 293 T cells. After transfection for 48 hours, the cells were lysed. After centrifugation at 12000 × *g* for 1 minute, the supernatant was collected. A Dual-Luciferase® Reporter Assay System (E1910, Promega, Madison, WI, USA) was used to detect luciferase activity. To each cell sample, 100 *μ*L firefly luciferase working solution and 100 *μ*L Renilla luciferase working solution were added to detect the activity of firefly luciferase and Renilla luciferase. The ratio of firefly luciferase activity to Renilla luciferase activity was determined as the relative luciferase activity.

### 2.7. Flow Cytometry

CD4^+^ T cells were isolated from peripheral blood by flow cytometry. The isolated cells were cultured in RPMI 1640 medium (Procell) containing 2% FBS, 100 U/mL penicillin, and 100 U/mL streptomycin. Briefly, 5 mL of peripheral blood was collected from healthy volunteers. Lymphocytes were isolated by the addition of lymphocyte isolation solution (P8610, SolarBio). The prepared single cell suspension was closed to Fc (anti-CD16/32, 564219, BD Biosciences, Franklin Lakes, NJ, USA) terminal and cultured at 4°C for 15 minutes. The dead cells were excluded with a Live/Dead cell staining kit (L34963, Thermo Fisher Scientific, Waltham, Massachusetts, USA). Finally, FITC-CD4 antibody (1:50, FITC-65143, ProteinTech Group Inc.) was incubated at 4°C for 30 minutes. M2 macrophages were detected by flow cytometry. After the induced macrophages were cocultured with Cal27-CSC-sEVs (30 *μ*g) for 48 hours or after lentiviral transduction, macrophages were incubated with antibodies to CD206 (1:50, 550889, BD Bioscience) and CD11b (1:50, 746572, BD Bioscience) at 4°C for 30 minutes, then detected by flow cytometry.

Macrophages with different treatments were cocultured with CD4^+^ T cells (2 × 10^4^ cells/well) in six-well plates (3412, Corning Inc., Corning, N. Y., USA). At 24 hours after the macrophages had been seeded in six-well plates, CD4^+^ T cells were seeded in the transwell chamber above the macrophages. After 48 hours, CD4^+^ T cells were collected. The CD4^+^ T cells were fixed and permeabilized with Cytofix/Cytoperm (88-8823-88, Thermo Fisher Scientific), and the cells were stained with fluorescein-conjugated antibodies to cytokines (PE-interferon-gamma [IFN-*γ*], 554552, BD Bioscience). The proliferation of CD4^+^ T cells was detected by the carboxyfluorescein diacetate succinimidyl ester (CFSE, Beyotime) dilution method. CD4^+^ T cells were then stained with 1 *μ*mol/L CFSE dye and incubated at 37°C for 10 minutes. Dead cells were excluded with a Live/Dead cell staining kit. A flow cytometer (FACSCalibur, BD Bioscience) was used for analysis.

### 2.8. Western Blot Analysis

RIPA cell lysis buffer (P0013B, Beyotime) containing PMSF was added to lyse tissues, cells, and sEVs to extract total protein. A BCA kit (P0028, Beyotime) was used for protein concentration determination according to the manufacturer's instructions. The protein on the gel was transferred to a polyvinylidene fluoride membrane (1620177, Bio-Rad, Richmond, Cal., USA). The membrane was blocked at room temperature for 1 hour with 5% BSA and probed overnight with primary rabbit antibodies to the following: CD81 (ab232390, 1:1000), CD63 (ab134045, 1:1000), calnexin (ab133615, 1:2000), LAMC2 (BA3650, 1:1000), PI3K (A01517-2, 1:1000), AKT (A00024, 1:500), and phosphorylated- (p-) AKT (BM4762, 1:500) at 4°C. Afterward, the membrane was reprobed for 1 hour with horseradish peroxidase-tagged goat antirabbit IgG secondary antibodies (ab6721, 1:5000, Abcam) at room temperature. The membrane was immersed in electrogenerated chemiluminescence reaction solution (1705062, Beyotime) for 1 minute at room temperature. Strip exposure imaging was performed with an ImageQuant LAS 4000 C instrument (General Electric Company, Schenectady, NY, USA). GAPDH (A01021, 1:5000, rabbit, Abbkine, Wuhan, China) was used to normalize target protein expression.

### 2.9. Reverse Transcription-Quantitative Polymerase Chain Reaction (RT-qPCR)

After total RNA was extracted with TRIzol (16096020, Thermo Fisher Scientific), the purity and concentration of the RNA were evaluated according to the absorbance at 260 and 280 nm, measured by spectrophotometry. The A260/A280 ratio of the sample was ≥ 1.8. For mRNA detection, a reverse transcription kit (RR047A, Takara, Kyoto, Japan) was used to reverse transcribe mRNA to obtain cDNA. For miRNA, a Poly(A) Tailing detection kit (B532451, Sangon Biotech, Shanghai, China) was used (including Universal PCR primer R and U6 Universal PCR primer R) and the cDNA of miRNAs containing poly(A) tails was obtained. PCR was performed with a LightCycler 480 instrument and SYBR Green I Master Mix, and the results were normalized to U6 and GAPDH. The 2^−ΔΔCT^ method was used to determine the ratios of target gene expression between the experimental group and the control group. The primer sequences are shown in Supplementary Table S2.

### 2.10. Transwell Assays

A transwell chamber (8 mm aperture; 3422, Corning) was used to detect cell migration *in vitro* in 24-well plates. First, 600 mL DMEM with 20% FBS was added in the lower chamber and equilibrated at 37°C for 1 hour. Cal27 cells with different treatments were resuspended in DMEM without FBS. Then, 3 × 10^5^ cells/mL cells were seeded into the chamber and cultured at 37°C and 5% CO2 for 24 hours. After the transwell insert was removed, the cells in the chamber were fixed with 4% paraformaldehyde for 20 minutes and stained with 0.1% crystal violet for 10 minutes. The surface cells were removed with a cotton ball and observed under an inverted fluorescence microscope (TE2000, Nikon, Tokyo, Japan). Five fields of vision were read randomly and photographed. The number of cells that passed through the chamber was counted. The average value was the number of cells passing through the chamber in each group. For cell invasion tests, the transwell chamber was precoated with Matrigel (356234, Becton, Dickinson and Company, Franklin Lakes, NJ, USA) to simulate the cell matrix. Three duplicate wells were prepared for each group.

### 2.11. Tumor Xenograft Model

All experimental procedures were approved by the Animal Ethics Committee of Shanghai Ninth People's Hospital, in compliance with the ARRIVE guidelines and the National Institutes of Health Guide for the Care and Use of Laboratory Animals (NIH Publications No. 8023, revised 1978). BALB/C adult male nude mice 6 weeks of age were reared under specific-pathogen-free conditions and given free access to drinking and food. The mice were randomly divided into three groups with six mice each: the oe-NC+sh-NC group, the oe-UCA1+sh-NC group, and the oe-UCA1+sh-LAMC2 group. Cal27 cells and macrophages treated with different Cal27-CSC-sEVs were suspended in PBS and implanted into the right axillary subcutaneous tissue in nude mice (1 × 10^6^ cells per mouse). Cal27-CSC-sEVs (5 *μ*g/mL) were injected into the caudal vein every 3 days after implantation. At 10 days after Cal27 cell inoculation, T cells (1 × 10^6^ cells per mouse) were injected into the peritoneal cavity in the mice. After 28 days, the nude mice were euthanized and the tumor weight and volume were measured. The tumor size was measured with Vernier calipers every 2 days. Tumor volume was calculated from three vertical measurements. After the mice were euthanized with pentobarbital sodium at 50 mg/kg (57-33-0, Shanghai Beizhuo Biotechnology Co., Ltd., Shanghai, China), the tumors were removed and photographed. The spleen was isolated into single cells and analyzed by flow cytometry.

### 2.12. Statistical Analysis

SPSS 21.0 (IBM Corp. Armonk, NY, USA) was used for statistical analysis. The measurement data are summarized as the mean ± standard deviation. Independent sample *t*-test was used for comparisons between groups. One-way analysis of variance (ANOVA) was applied for comparisons among multiple groups, and repeated measures ANOVA, followed by Tukey's post hoc test, was used to compare data at different time points. *p* < 0.05 was considered to indicate a statistically significant difference.

## 3. Results

### 3.1. OSCC-CSC-sEVs Promote M2 Polarization of Macrophages

**Figure 1:**
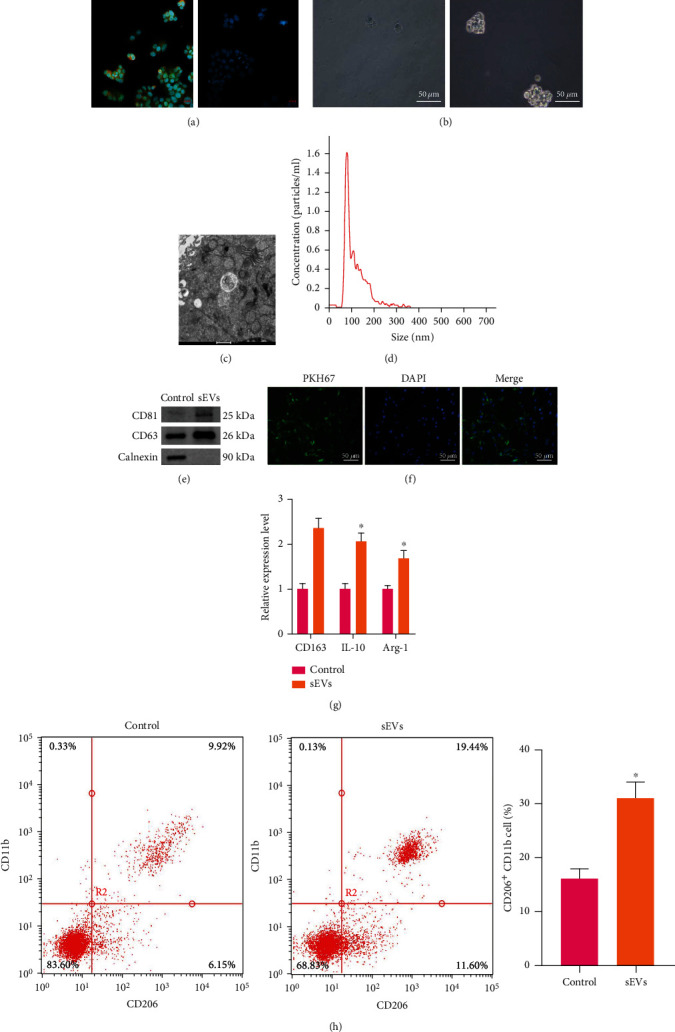
OSCC-CSC-derived sEVs (OSCC-CSC-sEVs) promote M2 polarization of macrophages. (a) CD133^+^CD44^+^ cell immunofluorescence staining synthesis map (left panel) and CD133^−^CD44^−^ cell immunofluorescence staining synthesis map (right panel, 500×). (b) The formation of spheres, observed under an optical microscope (200×). (c) The sEVs derived from OSCC-CSCs, observed under a transmission electron microscope (10000×). (d) Determination of exosome diameter by nanoparticle tracking analysis. (e) Western blot analysis detecting the exosome surface markers CD81 and CD63. (f) The uptake of sEVs by macrophages, observed under a confocal fluorescence microscope (200×). (g) Detection of M2 macrophage markers (CD163, IL-10, and Arg-1) by RT-qPCR after coculture of macrophages and OSCC-CSC-sEVs. (h) Detection of CD206^+^CD11b^+^ M2 macrophages by flow cytometry after coculture of macrophages and OSCC-CSC-sEVs. ^∗^*p* < 0.05. All cell experiments were repeated three times.

First, OSCC-CSCs were sorted using magnetic bead sorting technology. Fluorescence microscopy (Figure 1(a)) showed that the sorted CD133^+^CD44^+^ cells emitted red fluorescence and green fluorescence, thus indicating that CD133 and CD44 were highly expressed in OSCC cells. However, no red or green fluorescence was observed in CD133^−^CD44^−^ cells, thus suggesting that CD133 and CD44 were not expressed in those cells. The results of sphere-forming assays *in vitro* indicated that the ability of isolated OSCC-CSCs to form spheres was significantly stronger than that of OSCC cells (Figure 1(b)).

Second, OSCC-CSC-sEVs were successfully extracted. OSCC-CSCs were cultured *in vitro*, and sEVs were isolated from the supernatant. Transmission electron microscopy indicated that the isolated sEVs had typical sEV morphology with a generally consistent round- or oval-shaped membranous vesicle pattern (Figure 1(c)). NTA indicated that the diameters of sEVs ranged from 85 nm to 130 nm (Figure 1(d)). Western blot analysis indicated that the sEV surface markers CD63 and CD9 were highly expressed, but calnexin was not expressed on the surfaces of sEVs (Figure 1(e)).

Third, OSCC-CSC-sEVs were taken up by M*φ* and further promoted M2 polarization. PMA was used to induce THP-1 cells to differentiate into M*φ*. PKH67- (green-) labeled OSCC-CSC-sEVs were then cocultured with M*φ* for 24 hours, and the uptake of PKH67 by M*φ* was observed under a fluorescence microscope (Figure 1(f)). The results indicated that M*φ* successfully took up sEVs. Furthermore, RT-qPCR revealed that the expression of M2 macrophage markers [CD163, interleukin- (IL-) 10, and arginase-1 (Arg-1)] markedly increased in M*φ* cocultured with OSCC-CSC-sEVs (Figure 1(g)). Consistently, flow cytometry revealed a substantially greater proportion of CD206^+^CD11b^+^ cells in M*φ* cocultured with OSCC-CSC-sEVs (Figure 1(h)).

### 3.2. UCA1 Modulates the LAMC2/PI3K/AKT Axis in OSCC-CSCs via Binding miR-134

**Figure 2:**
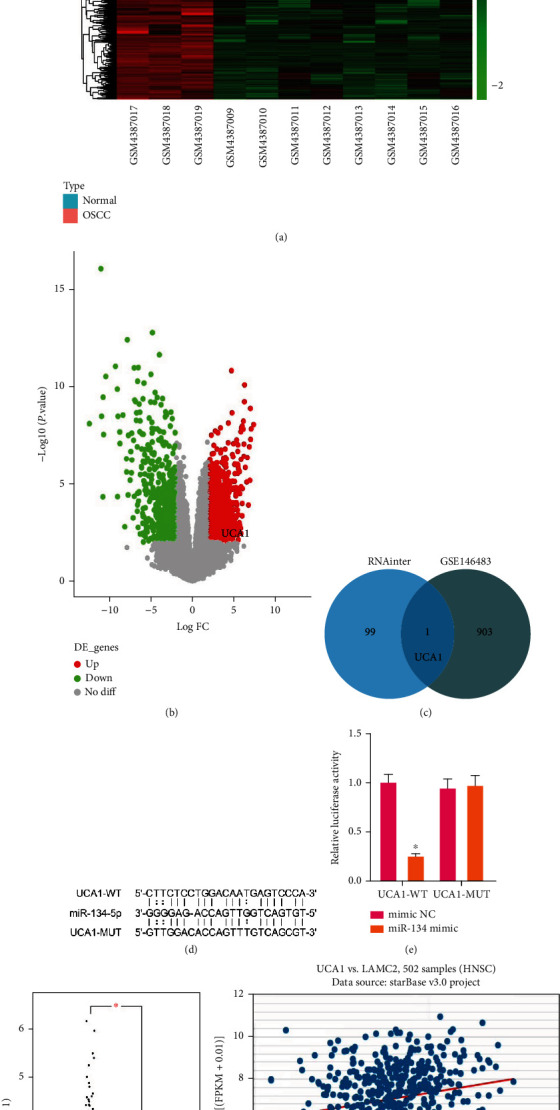
UCA1 modulates the LAMC2/PI3K/AKT axis in OSCC-CSCs via binding miR-134. (a) Heat map of differentially expressed genes among three normal tissues and eight OSCC tissues in the GSE146483 dataset. (b) Volcano map of differentially expressed genes among three normal tissues and eight OSCC tissues in the GSE146483 dataset. (c) Venn diagram of the intersection between lncRNAs binding miR-134 in the RNAInter database and downregulated lncRNAs in OSCC in GSE146483. (d) The binding sites of UCA1 and miR-134. (e) Dual-luciferase reporter assays detecting the binding relationship between UCA1 and miR-134. (f) The expression of UCA1 in the TCGA dataset, analyzed with the GEPIA database (red represents OSCC tissues, *n* = 519; black represents normal tissues, *n* = 44). (g) The correlation between UCA1 and LAMC2 expression in HNSCC samples (*n* = 502) from the TCGA dataset according to starBase database analysis. ^∗^*p* < 0.05, ^#^*p* > 0.05. All cell experiments were repeated three times.

We previously reported that miR-134 influenced the biological behavior of OSCC-CSCs via downregulation of the PI3K/AKT signaling pathway and inhibition of LAMC2 expression [15]. lncRNAs are widely recognized as ceRNAs that competitively bind miRNAs. To identify the lncRNAs that competitively inhibit miR-134 in OSCC, we predicted the lncRNAs that might bind miR-134 according to the RNAInter database and intersected the results with the upregulated lncRNAs in GSE146483; the results indicated that UCA1 binds miR-134 and is highly expressed in OSCC (Figures 2(a)–2(d)). Dual-luciferase reporter assays revealed that a miR-134 mimic significantly decreased the luciferase activity of UCA1-WT, but did not affect the luciferase activity of UCA1-MUT (Figure 2(e)). Hence, UCA1 binds to miR-134. According to the GEPIA database, UCA1 has significantly higher expression in OSCC than normal tissue (Figure 2(f)). In addition, the results of GO and KEGG analyses showed that miR-134 downregulates LAMC2 in OSCC and subsequently regulates the PI3K/AKT pathway (Supplementary Figure S1). According to starBase database analysis, the positive correlation between UCA1 and LAMC2 expression in OSCC was significant (Figure 2(g)). On the basis of these findings and our previous study [15], we speculated that UCA1 might affect the biological behavior of OSCC-CSCs by regulating the LAMC2/PI3K/AKT axis via binding miR-134.

### 3.3. UCA1 Modulates the PI3K/AKT Pathway in Macrophages via Targeting LAMC2

**Figure 3:**
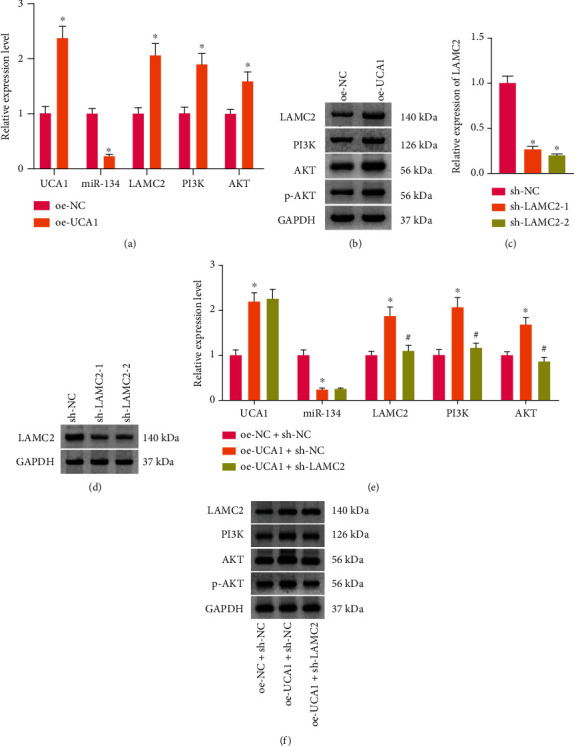
UCA1 modulates the PI3K/AKT pathway in macrophages via targeting LAMC2. (a) The expression of UCA1, miR-134, LAMC2, PI3K, and AKT in macrophages after overexpression- (oe-) UCA1 treatment, measured by RT-qPCR. (b) The protein expression of LAMC2, PI3K, AKT, and p-AKT in macrophages after oe-UCA1 treatment, detected by Western blot analysis. The silencing efficiency of sh-LAMC2, verified by RT-qPCR (c) and Western blot analysis (d). (e) The expression of UCA1, miR-134, LAMC2, PI3K, and AKT in macrophages after oe-UCA1 and sh-LAMC2 treatment, determined by RT-qPCR. (f) The protein expression of LAMC2, PI3K, AKT, and p-AKT in macrophages, determined by Western blotting. ^∗^*p* < 0.05, ^#^*p* > 0.05. All cell experiments were repeated three times.

First, we performed oe-UCA1 treatment in M*φ* through lentiviral transduction. RT-qPCR showed that the expression of UCA1, LAMC2, PI3K, and AKT significantly increased, whereas miR-134 expression decreased in M*φ* after treatment (Figure 3(a)). Western blot analysis verified that the expression of LAMC2, PI3K, AKT, and p-AKT significantly increased in M*φ* after treatment (Figure 3(b)). Second, we performed oe-UCA1 plus sh-LAMC2 treatment in M*φ*. The silencing efficiency of sh-LAMC2 was verified by RT-qPCR (Figure 3(c)) and Western blot (Figure 3(d)) analysis; sh-LAMC2-2 was used in subsequent experiments because it had the better silencing effect. RT-qPCR showed that the expression of LAMC2, PI3K, and AKT significantly decreased, whereas the UCA1 and miR-134 expression did not significantly change in M*φ* after oe-UCA1 plus sh-LAMC2 treatment (Figure 3(e)). Western blot analysis verified that the expression of LAMC2, PI3K, AKT, and p-AKT significantly decreased after oe-UCA1 plus sh-LAMC2 treatment (Figure 3(f)). Hence, UCA1 modulates the PI3K/AKT pathway via targeting LAMC2 in macrophages.

### 3.4. OSCC-CSC-sEVs Promote M2 Polarization of Macrophages by Transferring UCA1 and Targeting LAMC2

**Figure 4:**
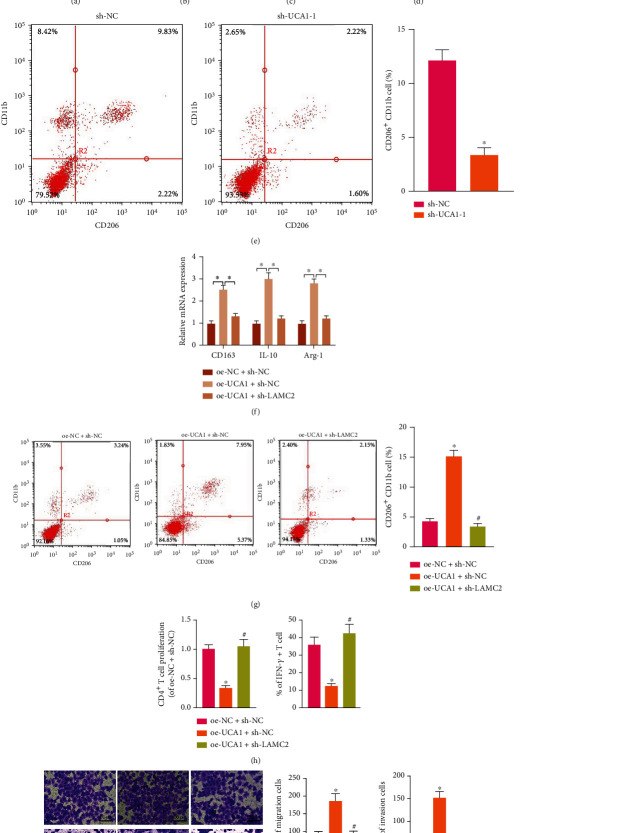
OSCC-CSC-derived sEVs (OSCC-CSC-sEVs) promote M2 polarization of macrophages by transferring UCA1 and targeting LAMC2. (a) UCA1 expression in Cal27-sEVs and Cal27-CSC-sEVs, measured by RT-qPCR. (b) Detection of UCA1 silencing efficiency, determined by RT-qPCR. (c) UCA1 expression in sEVs from Cal27-CSCs treated with sh-UCA1, detected by RT-qPCR. (d) RT-qPCR determination of the expression of the UCA1 and M2 macrophage markers CD163, IL-10, and Arg-1 in macrophages cocultured with sEVs from Cal27-CSCs treated with sh-UCA1. (e) Detection of M2 macrophages after macrophage coculture with sEVs from Cal27-CSCs treated with sh-UCA1, determined by flow cytometry. (f) The expression of M2 macrophage markers (CD163, IL-10, and Arg-1) in macrophages after oe-UCA1 and sh-LAMC2 treatment, determined by RT-qPCR. (g) Detection of CD206^+^CD11b^+^ M2 macrophages after oe-UCA1 and sh-LAMC2 treatment, determined by flow cytometry. (h) The proliferation number of CD4^+^ T cells and the proportion of IFN-*γ*^+^ T cells after coculture with macrophages treated with oe-UCA1 and sh-LAMC2, detected by flow cytometry. (i) Transwell assay detection of the migration and invasion ability of Cal27 cells cocultured with macrophages treated with oe-UCA1 and sh-LAMC2 (100×). ^∗^*p* < 0.05, ^#^*p* > 0.05. All cell experiments were repeated three times.

To investigate whether UCA1 might be delivered by OSCC-CSC-sEVs, we found that UCA1 was significantly more highly expressed in Cal27-CSC-sEVs than Cal27-sEVs on the basis of RT-qPCR (Figure 4(a)). After UCA1 was silenced in Cal27-CSCs by sh-UCA1-1 or sh-UCA1-2, the UCA1 expression significantly decreased according to RT-qPCR; sh-UCA1-2 was used in subsequent experiments because it had the better silencing effect (Figure 4(b)). UCA1 expression significantly decreased in Cal27-CSC-sEVs treated with sh-UCA1 (Figure 4(c)).

To investigate the effect of UCA1 on the M2 polarization of macrophages, we cocultured Cal27-CSC-sEVs and M*φ* macrophages. According to RT-qPCR, the expression of UCA1 and M2 macrophage markers (CD163, IL-10, and Arg-1) in macrophages decreased after coculture with sEVs from Cal27-CSCs treated with sh-UCA1 (Figure 4(d)). According to the results of flow cytometry, the proportion of CD206^+^CD11b^+^ cells was clearly diminished by coculture with sEVs from Cal27-CSCs treated with sh-UCA1 (Figure 4(e)). Therefore, OSCC-CSC-sEVs promote macrophage M2 polarization by transferring UCA1.

To further investigate the effect of UCA1 on M2 polarization by targeting LAMC2, we performed oe-UCA1 plus sh-LAMC2 treatment in Cal27-CSC-sEVs cocultured with M*φ*. RT-qPCR showed that the expression of CD163, IL-10, and Arg-1 significantly increased after oe-UCA1 treatment, but these effects were negated by additional sh-LAMC2 treatment (Figure 4(f)). Consistently, flow cytometry assays showed that the proportion of CD206^+^CD11b^+^ cells was significantly elevated after oe-UCA1 treatment, but this increase was abrogated by additional sh-LAMC2 treatment (Figure 4(g)).

We further investigated the LAMC2-mediated effects of UCA1, delivered by OSCC-CSC-sEVs, on the biological function of OSCC Cal27 cells and M*φ*. Differently treated M*φ* was cocultured with Cal27 cells. Transwell assays revealed that the migration and invasion of Cal27 cells were enhanced after oe-UCA1 treatment, but these effects were nullified by additional sh-LAMC2 treatment (Figure 4(i)). Differently treated M*φ* was cocultured with CD4^+^ T cells. Flow cytometry assays revealed that the proliferative number of CD4^+^ T cells and the proportion of IFN-*γ*^+^CD4^+^ T cells significantly decreased after oe-UCA1 treatment, and this response was augmented by the addition of sh-LAMC2 treatment (Figure 4(h)).

### 3.5. OSCC-CSC-sEVs Transferring UCA1 Accelerate M2 Macrophage Polarization and Enhance the Tumorigenicity of OSCC Xenografts in Nude Mice

**Figure 5:**
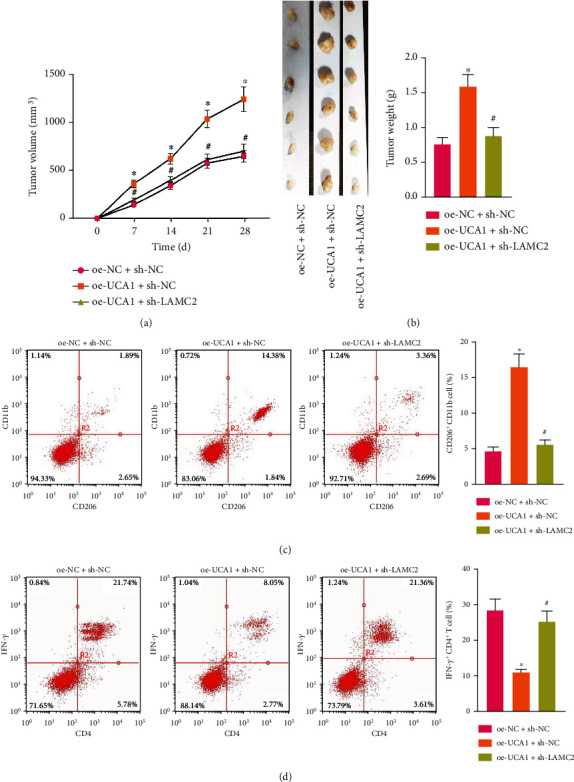
OSCC-CSC-derived sEVs transferring UCA1 accelerate M2 macrophage polarization and enhance the tumorigenicity of OSCC xenografts in nude mice. (a) Tumor volume curve and (b) tumor weight of nude mice (*n* = 6) in different treatment groups. (c) Detection of CD206^+^CD11b^+^ M2 macrophages in the tumor tissues of nude mice, determined by flow cytometry in different treatment groups. (d) Detection of IFN-*γ*^+^CD4^+^ T cells among the spleen cells in nude mice, determined by flow cytometry in different treatment groups. ^∗^*p* < 0.05, ^#^*p* > 0.05. The data in the figure were measurement data from 6 mice, which summarized as mean ± standard deviation.

The effects of UCA1 delivered by OSCC-CSC-sEVs on the tumorigenicity and immune function of OSCC cells in nude mice were further studied. Macrophages cocultured with Cal27 cells were implanted subcutaneously in nude mice. After implantation, different groups of OSCC-CSC-sEVs were injected via the tail vein every 3 days. At 10 days after Cal27 cell inoculation, T cells were injected into the peritoneal cavity in the mice. After 28 days, the nude mice were euthanized and the tumor weight and volume were measured. After oe-UCA1 treatment, the tumor volume (Figure 5(a)) and tumor weight (Figure 5(b)) in mice had clearly increased, but this effect was abrogated by sh-LAMC2.

**Figure 6:**
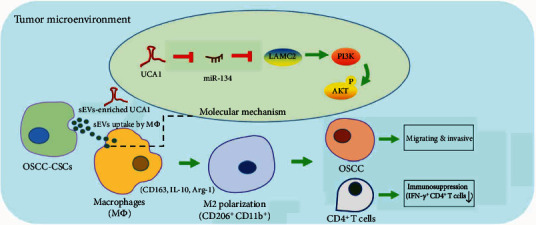
Schematic overview of the mechanism. OSCC-CSC-derived small extracellular vesicles (sEVs) transfer lncRNA UCA1, thus promoting M2 macrophage polarization via a LAMC2-mediated PI3K/AKT axis and further enabling cell migration and invasion of OSCC and modulating immunosuppression, partly by inhibiting CD4^+^ T-cell proliferation and IFN-*γ* production.

Flow cytometry indicated that the proportion of CD 206^+^CD11b^+^ cells (Figure 5(c)) increased and that of IFN-*γ*^+^CD4^+^ T cells (Figure 5(d)) decreased significantly after treatment with oe-UCA1, but this response was counteracted by additional sh-LAMC2 treatment. Therefore, OSCC-CSC-sEVs promote macrophage M2 polarization by transferring UCA1 and targeting LAMC2, thus enhancing the tumorigenicity and immunosuppression of OSCC in nude mice. Together, our results revealed that OSCC-CSC-sEVs transferring the lncRNA UCA1 promote M2 macrophage polarization via a LAMC2-mediated PI3K/AKT axis and further modulate immunosuppression, partly by inhibiting CD4+ T-cell proliferation and IFN-*γ* production (Figure 6).

## 4. Discussion

TAMs and associated sEVs play important roles in mediating intercellular communication, modulating immunosuppression, and facilitating cancer progression in the tumor microenvironment by transferring RNAs [3, 6]. Earlier studies have investigated cancer cell-derived sEVs, which promote M2 macrophage polarization in various cancers by transferring noncoding RNAs [6–8]. However, whether CSC-sEVs transfer noncoding RNAs, thus inducing M2 macrophage polarization, was largely unknown in most cancers [9]. In this study, we revealed that OSCC-CSC-sEVs transferring the lncRNA UCA1 promote M2 macrophage polarization via a LAMC2-mediated PI3K/AKT axis, thereby facilitating tumor progression and immunosuppression.

The lncRNA UCA1 has been reported to be involved in malignant progression of OSCC by targeting miR-143 [23] and miR-124 [24]. Furthermore, pancreatic cancer-derived exosomal UCA1 has been found to promote tumor angiogenesis by targeting miR-96 [25]; exosomal UCA1 modulates cervical cancer stem cell self-renewal and differentiation by targeting miR-122 [26]. However, the association between UCA1 and TAMs in OSCC was unknown. We found that UCA1, delivered by OSCC-CSC-sEVs, contributed to the migration and invasion of OSCC by binding to miR-134. Moreover, several reports have shown that OSCC-derived exosomes, by transferring proteins and miRNAs, promote M2 macrophage polarization [8, 27, 28]. Pang et al. have reported that OSCC-secreted exosomal CMTM6 induces M2 macrophages polarization [27]. Yuan et al. have reported that head and neck cancer cell-released exosomal PD-L1 facilitates M2 macrophage polarization [28]. Cai et al. have reported that OSCC-derived exosomes deliver miR-29a, thereby promoting M2 macrophage polarization [8]. However, the association between UCA1 and macrophages in OSCC was unknown. We demonstrated that UCA1 delivered by OSCC-CSC-sEVs promotes M2 macrophage polarization.

LAMC2 (laminin gamma 2 chain) has been reported to be involved in the malignant behavior of OSCC by targeting relevant miRNAs and lncRNAs [15, 29, 30]. Furthermore, LAMC2 expression is positively associated with macrophage infiltration in lung cancer [21]. LAMC2, via exosomal miR-146a, mediates increased chemotherapy sensitivity of ovarian cancer cells through the PI3K/AKT pathway [22]. Moreover, PI3K/AKT signaling is an important pathway for the biological behavior of cancer-derived sEVs and TAMs [16–20]. For instance, M2 macrophage-derived exosomal miRNAs inhibit cell migration and invasion in gliomas through the PI3K/AKT pathway [20]. However, the association between LAMC2 and sEVs or TAMs in OSCC was unknown. In a previous study [15], we found that LAMC2 participates in mediating OSCC-CSC-derived exosomal UCA1, thus promoting M2 macrophage polarization via the PI3K/AKT pathway.

M2-tumor-associated macrophages (M2-TAMs) are among the most abundant immunosuppressive cell types in the tumor microenvironment. M2 macrophage polarization further contributes to immunosuppression via inhibiting T-cell proliferation and the production of relevant cytokines [31–33]. In this study, we revealed that OSCC-CSC-derived exosomal UCA1, by targeting LAMC2, promotes M2 macrophage polarization and inhibits CD4^+^ T-cell proliferation and IFN-*γ* production *in vitro* and *in vivo*. Importantly, we demonstrated that M2-TAMs, by transferring exosomal UCA1 and targeting LAMC2, enhance cell migration and invasion of OSCC *in vitro* and the tumorigenicity of OSCC in nude mice *in vivo*. Furthermore, increasing evidence indicates that tumor-derived exosomes and M2-TAMs play roles in the malignant biological behavior of OSCC [34, 35]. However, their immunosuppressive mechanisms and functional roles in OSCC microenvironment remain to be further explored [2].

## 5. Conclusions

In summary, our data suggested that the lncRNA UCA1 increased in OSCC-CSC-derived sEVs, and these UCA1-rich CSC-secreted sEVs were transferred to unpolarized macrophages and induce macrophage polarization toward protumor-related M2 macrophages by targeting LAMC2 via the PI3K/AKT pathway. These findings indicated that the OSCC-CSCs use sEV-transferring UCA1 to modulate the immunosuppressive microenvironment, thus enabling cell migration and invasion of OSCC and enhancing tumorigenicity. Our findings provide a new understanding of the molecular mechanism of OSCC-CSC and suggest a potential therapeutic strategy for OSCC by targeting CSC-sEVs and M2-TAMs.

## Data Availability

The data used to support the findings of this study are included within the article.

## Supplementary Materials

Supplementary 1 Figure S1: KEGG analysis of miR-134 and LAMC2-regulated PI3K/AKT pathway. A, Enriched KEGG analysis terms of miR-134-regulated pathways. B, LAMC2-regulated PI3K/AKT pathways.

Supplementary 2 Table S1: the primer sequence of short hairpin- (sh-) RNA lentivirus. Table S2: the primer sequence of RT-qPCR.
