# Supplementary material for: Oral Cancer Stem Cell-Derived Small Extracellular Vesicles Promote M2 Macrophage Polarization and Suppress CD4+ T-Cell Activity by Transferring UCA1 and Targeting LAMC2
Source: Stem Cells Int. 2022 Nov 28;2022:5817684. doi: 10.1155/2022/5817684 (PMC9723417; doi:10.1155/2022/5817684)
Supplement: Supplementary 1 — Figure S1: KEGG analysis of miR-134 and LAMC2-regulated PI3K/AKT pathway. A, Enriched KEGG analysis terms of miR-134-regulated pathways. B, LAMC2-regulated PI3K/AKT pathways. [file 5817684.f1.docx]

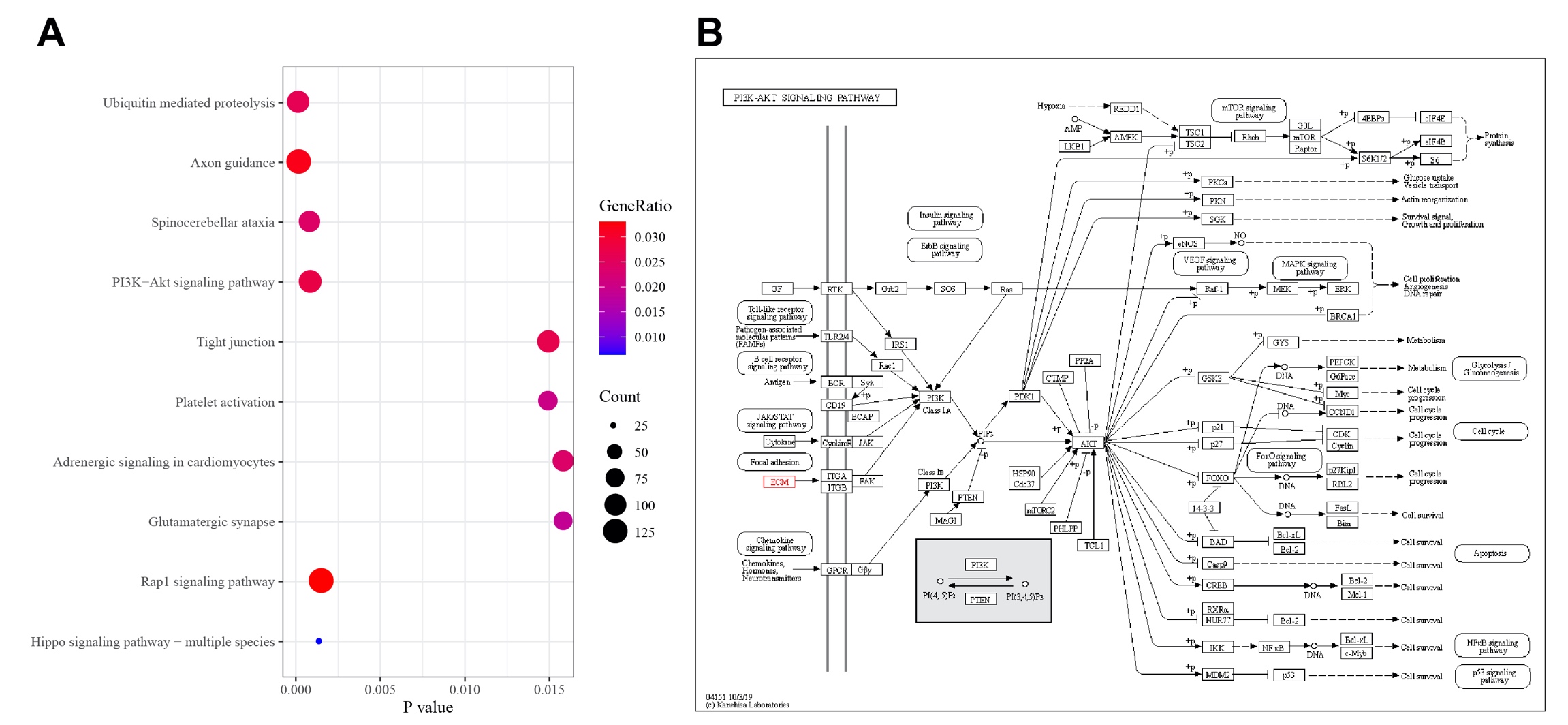


**Supplementary Figure S1**. KEGG analysis of miR-134 and LAMC2 regulated PI3K/AKT pathway. A, Enriched KEGG analysis terms of miR-134-regulated pathways. B, LAMC2-regulated PI3K/AKT pathways.
