# Supplementary material for: Oral Cancer Stem Cell-Derived Small Extracellular Vesicles Promote M2 Macrophage Polarization and Suppress CD4+ T-Cell Activity by Transferring UCA1 and Targeting LAMC2
Source: Stem Cells Int. 2022 Nov 28;2022:5817684. doi: 10.1155/2022/5817684 (PMC9723417; doi:10.1155/2022/5817684)
Supplement: Supplementary 2 — Table S1: the primer sequence of short hairpin- (sh-) RNA lentivirus. Table S2: the primer sequence of RT-qPCR. [file 5817684.f2.docx]

**Supplementary Table S1** The primer sequence of short hairpin (sh)-RNA lentivirus

| Name | Sequence |
| --- | --- |
| sh-negative control for Cal27-CSCs | 5’-TTCTCCGAACGTGTCACGT-3’ |
| sh-UCA1-1 for Cal27-CSCs | 5’-GUGAAGACAAUCAACUCAAUU-3’ |
| sh-UCA1-2 for Cal27-CSCs | 5’-GAGCCGAUCAGACAAACAAUU-3’ |
| sh-negative control for Mφ | 5’-TTCTCCGAACGTGTCACGT-3’ |
| sh-LAMC2-1 for Mφ | 5’-GCAGAATACAGTGTCCATA-3’ |
| sh-LAMC2-2 for Mφ | 5’-GCAGGTGTTTGAAGTGTAT-3’ |

**Supplementary Table S2** The primer sequence of RT-qPCR

| Name | Primer sequence (5’-3’) |
| --- | --- |
| UCA1 | F: TTCCACATATTTGGCAACCAGAC |
|  | R: GATTAAGCTGAGGCTGGCAAAG |
| miR-134 | F: AACTGCAGAGCTGTGGTTCTGT |
|  | R: CGCGGATCCCGTGTCATCGCA |
| LAMC2 | F: GACAAACTGGTAATGGATTCCGC |
|  | R: TTCTCTGTGCCGGTAAAAGCC |
| PI3K | F: TGTAGTGGTGGACGGCGAAGTA |
|  | R: GGGAGGTGTGTTGGTAATGTAGCA |
| AKT | F: TGGGTTCAGAAGAGGGGAGAA |
|  | R: AGGGGATAAGGTAAGTCCACATC |
| CD163 | F: AGCAGACTACTCCAACATCC |
|  | R: TGGCACAGTTGTCTCTATCC |
| IL-10 | F: GCCACCCTGATGTCTCAGTT |
|  | R: GTGGAGCAGGTGAAGAATGC |
| Arg-1 | F: TGGA CAGACTAGGAATTGGCA |
|  | R: CCAGTCCGTCAACATCAAAACT |
| U6 | F: AAAGCAAATCATCGGACGACC  R: GTACAACACATTGTTTCCTCGGA |
| GAPDH | F: ACATCGCTCAGACACCATG |
|  | R: TGTAGTTGAGGTCAATGAAGGG |
